# Adolescents with Mild Stunting Show Alterations in Glucose and Insulin Metabolism

**DOI:** 10.1155/2010/943070

**Published:** 2010-12-29

**Authors:** Carla Danusa da Luz Santos, Ana Paula Grotti Clemente, Vinicius José Baccin Martins, Maria Paula Albuquerque, Ana Lydia Sawaya

**Affiliations:** ^1^Department of Physiology, Federal University of São Paulo, São Paulo, 04023-062 SP, Brazil; ^2^Nutrition Education and Recovery Center, São Paulo, Brazil

## Abstract

*Purpose*. To evaluate glucose and insulin profiles in adolescents with mild stunting and overweight in order to assess the possibility of increased predisposition to diabetes. *Subjects and Methods*. The study population consisted of 66 pubertal adolescents classified as mildly stunted (height-for-age z scores ≥−2 and <−1) or of normal stature, as well as overweight (body mass index ≥85th percentile) or normal weight. Beta-cell function and insulin resistance were evaluated according to the homeostasis model assessment (HOMA). *Results*. In the group with mild stunting, glucose, insulin, and HOMA-IR levels were significantly higher in overweight adolescents compared with those of normal weight, whereas HOMA-B levels were significantly lower. Adolescents with mild stunting showed significantly higher accumulations of body and abdominal fat than their normal stature counterparts. *Conclusions*. The presence of mild stunting was associated with higher levels of glucose and insulin, diminished function of beta cells, and increased insulin resistance. These results reinforce the need for intervention in adolescents with mild stunting.

## 1. Introduction

Several studies have been done to show how nutritional deprivation during critical periods of development could cause long-lasting changes that lead to obesity and associated co-morbidities in adulthood. Studies with individuals who were exposed to famine during the Dutch famine suggest that malnutrition in early pregnancy is associated with increased abdominal obesity in women [1], increased prevalence of coronary heart disease [2], more atherogenic lipid profile [3–5], having a preference for diet richer in fat and having lower physical activity score [4, 6]. These factors seem to be a consequence of adaptive processes developed during intrauterine life to ensure survival and persist throughout life. Alongside, the metabolism of glucose and insulin also changes in those who suffered nutritional deprivation in early life. Individuals with intrauterine growth retardation showed insulin resistance compared with a control group [7, 8]. However, physiological and metabolic mechanisms are not fully matured at birth and continue the maturation process in the immediate postnatal period [9]. Thus, unfavorable nutritional conditions early in life could cause the same metabolic changes observed in those who had prenatal malnutrition. Studies have shown, in fact, that low weight gain in infancy is also associated with coronary heart disease in adulthood independent of birth weight [10, 11]. Evidence also suggests that postnatal undernutrition can induce alterations in glucose metabolism generating a predisposition for diabetes. In this context, Gonzalez-Barranco [12] submitted a group of young adults, each of whom had a history of undernutrition in the first year of life, to the oral glucose tolerance test and detected elevated levels of glucose and insulin in comparison with the control group, independent of birth weight, body mass index (BMI), or age.

One way to identify the nutritional status during critical periods of prenatal and postnatal growth is the assessment of height [9]. A deficit of height for age is usually the result of a prolonged period of illness with frequent infections and malnutrition, or both [13]. 

Studies involving children with nutritionally induced stunting have shown that the function of pancreatic beta cells was diminished while insulin sensitivity was increased [14]. Recently, Florêncio and co-workers [15] reported that short stature represented a risk factor for alterations in lipid profile, insulin resistance, and abdominal obesity in adult women from slums in Maceió, north-eastern Brazil. Moreover, a longitudinal study comparing stunted and normal girls revealed that stunted individuals exhibited a greater susceptibility to gain weight in relation to height and to accumulate abdominal fat [16]. Elevated respiratory quotients (indicating high carbohydrate oxidation) and reduced lipid oxidation rates have also been observed in stunted boys and girls [17]. Additionally, stunted girls exhibited decreased total energy expenditure compared with normal girls [18], and this was considered to be a further factor in the predisposition to weight gain. All of these studies reinforce the hypothesis that stunting alters the regulation of physiological mechanisms that are responsible for energy conservation and fat accumulation, resulting in obesity in adult life with high risk of co-morbidities such as diabetes. 

Recently the World Health Organization (WHO) amended the classification of children and adolescents according to height-for-age Z score (HAZ). Whereas individuals exhibiting HAZ values between −2 and −1 SD were previously categorised as having mild stunting, and therefore with chronic undernutrition, the latest recommendation considers such individuals to be normal. The hypothesis tested in the present study was that these individuals present alterations in glucose and insulin metabolism and therefore should not be categorized as having normal nutritional status. Based on this proposition, this investigation aimed to determine whether pubertal individuals with mild stunting (<−1 and ≥−2  Z scores) presented alterations in glucose and insulin metabolism that were similar to those described for subjects with more severe stunting. If this hypothesis is correct, it would clearly be essential to consider more sensitive cutoff points in the classification of stature in order to allow an increased surveillance of the metabolic changes that occur in mild nutritional stunting.

## 2. Materials and Methods

The study was approved by the Committee of Ethics in Research of the Universidade Federal de São Paulo (UNIFESP; protocol no. 0617/06). All of the procedures employed complied with the ethical principles contained in the Declaration of Helsinki as stated by the World Medical Association. Written informed consent was obtained from the participants, or their parents or legal guardians where appropriate, prior to the commencement of the study.

### 2.1. Study Population

As part of a comprehensive investigation of the effects of mild undernutrition a cross-sectional study was done. A population of 66 pubertal adolescents aged between 10 and 19 years and attending government-funded schools and other institutions located in slums near to the campus of UNIFESP in the south side of the city of São Paulo were selected, out of a group of more 5000 students who were measured in a anthropometric census. Only pubertal adolescents were selected for the study as we wanted to investigate the effects of mild stunting in glucose and insulin metabolism after the subjects attained puberty and to avoid the confounding effects of pubertal stages in this hormone. Prior to the commencement of the project, all potential participants were submitted to a clinical and laboratory screening, which included blood, urine, and parasitological tests. Subjects presenting infectious or parasitic diseases were treated according to normal protocols at the Hospital São Paulo and were subsequently invited to take part in the study. Individuals diagnosed with endocrinopathies, neurological problems and/or dementia, cardiovascular, respiratory or metabolic disorders, those using corticosteroids and those presenting physical limitations were excluded from the study. Subjects were examined by a trained physician and classified with regard to sexual development according to Tanner [19]. Individuals who had attained the appropriate WHO cutoff points, namely, breast-stage 2 for girls and genitalia-stage 3 for boys, were considered to be at the beginning of the pubertal growth spurt [13]. A standard and validated questionnaire was used to collect information on socioeconomic and housing conditions [20].

### 2.2. Anthropometric Measurements

Anthropometric indices were computed with the aid of Epi Info software version 6.2 as the curves of the CDC 2000 were used to calculate nutritional status. The weight of each participant was obtained by single measurement using platform scales with a capacity of 150 kg and an accuracy of 10 g. Stature was assessed by a single trained operator according to standard procedures [21] using an AlturExata (TBW, São Paulo, Brazil) portable stadiometer with a precision to the nearest 0.1 cm. Screening measurements were carried out initially in the classroom with subjects dressed in school uniforms and were subsequently repeated with the participants wearing only their undergarments before body composition measurements at the hospital. This last weight measurement was considered for the analysis. BMI values were determined as the quotient between weight and height squared (kg/m^2^). Waist circumference was determined with the subjects in a standing position with abdomen relaxed and arms relaxed alongside the body. A flexible measuring tape (0.1 mm accuracy) was placed horizontally at the midpoint between the bottom edge of the last rib and the iliac crest, and measurements were taken with the tape firmly applied on the skin but without compression of tissues. 

Since the purpose of the study was to detect early changes occasioned by stunting, the sample population was classified according to HAZ, namely, stunted (HAZ <−1 SD and ≥−2 SD) and normal stature (HAZ >−1 SD). Participants were further categorised according to BMI-for-age percentiles as overweight (≥85th), normal (>5th and <85th) or underweight (≤5th) by comparison with standard reference values based on the Centers for Disease Control and Prevention (CDC) 2000 growth charts for children and adolescents in the United States [22].

### 2.3. Biochemical Analyses

Blood samples (20 mL) were collected by vein puncture and analysed for levels of plasma glucose (colorimetric method; Bayer Inc., New York, USA) and insulin without C peptide (enzyme immunoassay method; TOSOH Inc, Tokyo, Japan).

In the homeostasis model assessment (HOMA), indices are calculated using a nonlinear mathematical model to describe the balance between hepatic-produced glucose and basal fasting state insulin that is maintained through a feedback loop mechanism between the pancreatic beta cells and the liver [23]. HOMA indices can be readily derived from fasting glycaemia and insulinaemia and have been employed in numerous population-based investigations [24–26]. HOMA was employed in the present study to evaluate the function of pancreatic beta-cells (HOMA-B) and insulin resistance (HOMA-IR), and the respective indices were calculated according to the following equations [23–27]:

(1) $$\begin{array}{c} \text{HOMA-B}=\left[\frac{20\times \text{fasting}\;\text{insulin}\;\left(\text{mU}/\text{L}\right)}{\text{fasting}\;\text{glucose}\;\left(\text{mmol}/\text{L}\right)}\right]-3.5, \end{array}$$

(2) $$\begin{array}{c} \text{HOMA-IR}\;\;\;\;\;\;\;\;\;\;\;\;\;\;\;\;\;\;\\ \;=\frac{\text{fasting}\;\text{insulin}\;\left(\text{mU}/\text{L}\right)\times \text{fasting}\;\text{glucose}\;\left(\text{mmol}/\text{L}\right)}{22.5} \end{array}$$

## 3. Results

The socioeconomic characteristics of the population (Table 1) revealed that, although the families of the participants were poor, the mean daily per capita income was above the poverty level (generally considered to be in the region of US$1.25). However, illiteracy amongst mothers was quite high at around 11%, and a significant number of homes comprised inadequate shacks built wholly or partly with scrap wood.

Table 2 shows anthropometric characteristics of the study population. Normal stature/ overweight participants showed significantly higher mean weight and BMI values than their stunted counterparts but similar BMI-for-age percentiles. Within the stunted group, the levels of glucose, insulin and HOMA-IR were significantly higher in overweight subjects compared with those with normal weight (Table 3), whereas HOMA-B levels were significantly lower. Diversely, no significant differences were detected in HOMA-B and HOMA-IR between overweight and normal weight adolescents of normal stature, except for insulin levels that were much higher in overweight individuals. Stunted adolescents presented significantly higher accumulations of body and abdominal fat than their normal stature counterparts (Table 4).

## 4. Discussion

In this study we did not measure the stature of the parents to check for genetic similarities between their stature and that of participants, as it is known that in low socioeconomic groups growth potential may not be fully expressed and, therefore, parental stunting may arise as a result of the cumulative effect of poverty endured by several generations of the family [28, 29].

Although stunted adolescents exhibited lower weight and BMI absolute values, higher percentage of body fat and abdominal fat was found in comparison to normal stature individuals. Similar results were found in a study with African girls with moderate stunting (HAZ <−2) using skinfolds and waist circumference for body composition evaluation [30]. Additionally, a three-year followup study of pubertal individuals by Martins et al. [31] revealed that stunted individuals (HAZ <−1.5) exhibited higher accumulation of body fat and lower lean mass compared with normal stature subjects. The results of the present study showed, in a similar way, that the accumulations of body and abdominal fat were greater in stunted adolescents than in normal stature individuals, even though the HAZ cutoff point used (<−1 and ≥−2) was less stringent than in other studies [31]. 

It has been reported that children with nutritionally-induced stunting (HAZ <−1.5) who were not overweight exhibited diminished function of pancreatic beta-cells together with increased insulin sensitivity in comparison with normal stature children. It has been suggested that the increase in insulin sensitivity in these children may be due to a higher number of insulin peripheral receptors, especially in the adipose and muscle tissues. This increase in sensitivity could establish a counter-regulation mechanism to compensate for the low concentrations of plasma insulin [14]. In the present study, adolescents with mild stunting who were also overweight presented higher glucose, insulin and HOMA-IR levels, but lower HOMA-B index than their normal weight counterparts. These results show that in the presence of overweight, stunted individuals continue to show impaired beta-cell activity but now alongside with insulin resistance. These alterations indicate a higher risk of future diabetes in adolescents with mild stunting and, different from controls, the presence of overweight precipitated to insulin resistance. A previous study involving woman with short stature and overweight found similar increase in insulin resistance (HOMA IR) together with impaired glycaemic and lipid profiles, as BMI increased [15]. While an increase in total body mass was associated with a moderate decline in peripheral sensitivity to insulin, abdominal obesity was characterized by a steep decline in such sensitivity accompanied by a reduction in glucose peripheral stimulus and a reduction in insulin output. 

The cutoff point employed in the present study included subjects with mild stunting only (classified as normal stature individuals according to the new recommendations). The present results in insulin and glucose metabolism clearly indicate that the surveillance of such individuals must not be neglected since they have increased risk of diabetes in the future. 

The cutoff points employed in evaluating nutritional status have passed through several modifications over the years. The latest criteria, recommended by WHO in 2006, consider a cutoff point of −2 DP for the HAZ indicator to identify children and adolescents with chronic nutritional deprivation, while those above this point would be of normal stature and, therefore, of normal nutritional status [32]. This latest modification was carried out in order to eliminate the risks of false designation of undernutrition, thereby guaranteeing the identification of those children who are actually undernourished. On the other hand, it has recently been demonstrated that the risk of children with WAZ values in the range of −2 to −1 DP dying from diarrhea, pneumonia, and malaria is twofold higher than for children with WAZ values >−1 DP [33]. The new cutoffs, therefore, may not be adequate for the purposes of primary health care since they hamper the identification of children with mild undernutrition and may impede early intervention not only for short but also long-lasting consequences of undernutrition. On this basis, we suggest that the latest modification of WHO cutoffs should be revised.

## 5. Conclusions

Adolescents with mild stunting (considered with normal stature according to the latest classification) exhibited increased body fat, especially abdominal fat. And when mild stunting is associated with overweight, higher concentrations of glucose and insulin, as well as diminished function of betacells and increased insulin resistance, were found. All these changes are known to be associated with greater risk of developing chronic diseases. These findings provide evidence that the current recommendations need to be reassessed.

##  Author's Contributions

C. D. da Luz Santos and A. P. G. Clemente participated in the design of the study and the collection of data, and also codrafted the paper. V. J. B. Martins revised the paper. M. P. Albuquerque helped with the collection of data. A. L. Sawaya conceived the design of the study, coordinated its implementation, and also revised the final paper.

## Figures and Tables

**Table 1:** Socioeconomic characteristics of the study population.

Parameter	Value
Schooling (measured as percentage illiteracy)	
Mother	10.6%
Father	5.6%

Family size	
Average number of people per dwelling	6 ± 3.6

Income	
Monthly family income	US$ 484 ± 328.0
Daily per capita income	US$ 4 ± 2.7

Type of abode	
Wooden house	6.0%
Wooden + brick house	3.9%
Brick house	90.2%

**Table 2:** Anthropometric characteristics of the study population.

Parameters	Mild stunting^1^		Normal stature^2^	
	Mean	Standard deviation	Mean	Standard deviation
Normal weight^3^	*n* = 17		*n* = 22	

Height (cm)	149.04	8.97	164.87	9.90
Height-for-age z scores	−1.56	0.25	0.41*	1.00
Weight (kg)	42.82	10.58	53.55	8.64
Body mass index (kg/m^2^)	19.00	2.73	19.61	2.15
BMI-for-age percentile	43.09	28.37	47.02	25.76

Overweight^4^	*n* = 12		*n* = 15	

Height (cm)	147.45	4.92	158.85	9.59
Height-for-age *z* scores	−1.50	0.31	0.11*	0.75
Weight (kg)	54.00	6.25	73.26*	20.02
Body mass index (kg/m^2^)	24.76	1.76	28.53*	4.92
BMI-for-age percentile	91.19	3.51	94.87	3.62

In each row, significant differences between short and normal stature groups are shown by asterisk (Student *t*-test; *P* < .05)

^1^Height-for-age z scores <−1 and ≥−2.

^2^Height-for-age z scores ≥−1.

^3^BMI-for-age percentile > 5th and < 85th.

^4^BMI-for-age percentile ≥ 85th.

**Table 3:** Biochemical characteristics of the study population.

Parameters	Mild stunting^1^				Normal stature^2^			
	Normal weight^3^		Overweight^4^		Normal weight^3^		Overweight^4^	
	Mean	Standard error	Mean	Standard error	Mean	Standard error	Mean	Standard error
	*n* = 17		*n* = 12		*n* = 22		*n* = 15	
Glucose (mg/d)	86.22	0.94	90.17*	1.13	87.18	1.47	89.73	1.80
Insulin (U/ml)	7.65	0.94	13.18*	1.12	8.11	2.18	17.43*	2.66
HOMA-B (%)	82.92	1.12	78.18*	1.35	82.07	1.79	79.19	2.19
HOMA-IR (%)	0.71	0.01	0.75*	0.01	0.72	0.01	0.75	0.02

In each row, significant differences between normal and overweight groups are shown by asterisk (ANCOVA adjusted according to gender; *P* < .05).

^1^Height-for age z scores <−1 and ≥−2.

^2^Height-for age z scores ≥−1.

^3^BMI-for-age percentile > 5th and < 85th.

^4^BMI-for-age percentile ≥ 85th.

**Table 4:** Body composition of the study population.

Parameters	Mild stunting^1^		Normal stature^2^	
	Mean	Standard error	Mean	Standard error
	*n* = 9		*n* = 30	
Body fat (kg)	18.53	1.22	14.61∗	0.65
Body fat (%)	31.80	1.87	26.86∗	0.99
Lean mass (kg)	35.65	1.18	36.90	0.63
Abdominal fat (kg)	1.27	0.15	0.96	0.08
Abdominal fat (%)	32.83	2.30	26.23∗	1.22
Waist circumference (cm)	76.55	2.20	71.72	1.19

In each row, significant differences between short and normal groups are shown by asterisk (ANCOVA adjusted according to gender, age, and weight; *P* < .05).

^1^ Height-for-age z scores <−1 and ≥−2.

^2^ Height-for-age z scores ≥−1.
